# A multicenter cross-sectional study on factors associated with caregiving appraisal in pediatric acute leukemia caregivers

**DOI:** 10.1371/journal.pone.0324589

**Published:** 2025-06-06

**Authors:** Phoebe Abonyo Ouru, Hong Zheng, Zhi Lin, Minghua Yang

**Affiliations:** 1 Department of Pediatrics, The Third Xiangya Hospital, Central South University, Changsha, Hunan, China; 2 Hunan Clinical Research Center of Pediatric Cancer, The Third Xiangya Hospital, Central South University, Changsha, Hunan, China; Stamford Health System: Stamford Hospital, UNITED STATES OF AMERICA

## Abstract

**Introduction:**

Caring for pediatric acute leukemia (PAL) patients poses significant psychological challenges for family caregivers, impacting their well-being and caregiving capacity. This multicenter study aimed to assess caregivers’ perception of their caregiving role using the revised Caregiving Appraisal Scale (rCAS) and identify factors associated with negative and positive caregiving appraisals among caregivers of children with PAL.

**Methods:**

A cross-sectional survey was conducted across three hospitals affiliated with Central South University. The 4-domain rCAS assessed both positive (satisfaction, mastery) and negative (burden, environmental impact) caregiving appraisals. Patient clinical data as well as demographics of both patients and caregivers were used to perform univariate, correlation, and multivariate regression analyses, identifying predictors of caregiver psychological distress.

**Results:**

Out of 111 recruited caregivers, 101 valid responses were analyzed. Univariate subgroup analysis showed that several caregiver- and patient-related factors were significantly associated with increased caregiver burden and environmental impact. For mastery and satisfaction, some factors were associated with higher appraisal scores while others were related to significantly lower scores. Correlation analysis showed that patient discomfort and difficulties significantly correlated with both negative and positive appraisals.

The regression models revealed that the independent influencing factors contributed to about 23.9% of caregiver-perceived negative impact (*P* = 0.024) and 30.3% of caregiver-perceived positive impact (*P* < 0.001). Patient worry (*P* = 0.024), communication difficulties (*P* = 0.011), relationship to the patient (*P* = 0.017), and medical insurance type (*P* = 0.035) predicted negative appraisals while pain (*P* = 0.008), anxiety about surgical procedures (*P* = 0.022), relationship to the patient (*P* = 0.031), and caregiver educational level (*P* = 0.023) predicted positive appraisals.

**Conclusion:**

Caregivers of pediatric acute leukemia experience considerable psychological strain. Despite these challenges, some caregivers report significantly higher positive appraisals. Targeted interventions are needed to reduce caregiver burden and enhance positive caregiving outcomes.

## 1. Introduction

Pediatric leukemia, with over 95% being of the acute type, is the most prevalent and aggressive childhood cancer worldwide [1]. This life-threatening disease necessitates prolonged and intensive treatment protocols including chemotherapy and hematopoietic stem cell transplantation (HSCT) [2,3]. These rigorous treatment regimens place significant financial, emotional, and psychological demands on both patients and their families [4–6]. While therapeutic advancements have improved survival rates, caregiving responsibilities for children with Pediatric Acute Leukemia (PAL) continue to impose a substantial burden on family members, particularly primary caregivers [5], who are integral to managing the complexities of treatment, providing emotional support, and preserving the child’s quality of life throughout the demanding journey [7].

The psychological toll of caregiving in pediatric oncology is profound. Caregivers often experience high levels of stress, anxiety, and depression due to the emotional and physical demands of supporting a child through long-term treatment [8]. This toll can affect their mental health, well-being, and even long-term physical health. Research has shown that caregiving stress significantly predicts depressive symptoms and anxiety among parents, highlighting the importance of mental health support for caregivers [9]. Caregiving also leads to financial strain and social isolation, as caregivers may need to reduce working hours or forgo employment altogether to care for the child. Furthermore, studies suggest that caregivers’ quality of life at the end of treatment is linked to caregiving strain and cancer-related variables, underscoring the need for targeted support during this challenging period [10]. Therefore, understanding factors that are associated with caregiver-perceptions on caregiving is critical for informing strategies that enhance the well-being of both caregivers and the child’s care.

Despite the significant burdens placed on caregivers, there is a distinct lack of research focusing on caregiving dynamics within pediatric oncology, particularly in low- and middle-income countries (LMICs) [11,12], where caregiving burdens may be amplified due to resource limitations, healthcare disparities, and cultural factors [13] which may also apply to certain regions of China. Although China is an upper-middle-income country, it is still classified as part of LMICs. Additionally, there are significant regional disparities, particularly in rural areas, where caregiving dynamics and challenges may closely resemble those in other LMICs [10,14]. Therefore, research from LMICs provides a valuable indication to investigate the caregiving experience in China including lower-resource settings, to further understand caregiver burden and the need for targeted support across diverse healthcare settings.

The caregiving appraisal scale (CAS) offers a comprehensive framework for understanding the multifaceted experiences of caregivers, by assessing both the positive and negative dimensions [15]. Caregiving is known to have a profound impact on caregiver health outcomes including stress, emotional well-being, and long-term physical health [8]. Positive appraisals, such as caregiving satisfaction and mastery, are associated with enhanced resilience, whereas negative appraisals, including feelings of burden and environmental strain, contribute to psychological distress and can increase the risk of caregiver burnout [16]. Factors such as caregiver sex, age, employment status, and access to social support have been identified as important determinants of caregiving perceptions [11,17]. Although caregiving appraisal has been studied in the context of adult chronic illnesses, research on caregiving appraisal in pediatric oncology remains limited, particularly in the Chinese context.

This study aims to address this research gap by investigating the caregiving appraisals of family caregivers of pediatric acute leukemia patients in China, using the revised Caregiving Appraisal Scale (rCAS). Specifically, we seek to [1] explore the relationship between caregiver demographics (e.g., sex, education level, employment status) and caregiving appraisals, [2] assess how patient-related factors (e.g., prognosis, duration of treatment) influence caregivers’ perceptions, and [3] identify predictors of both positive and negative caregiving appraisals. By exploring both the positive and negative aspects of caregiving appraisal, this study seeks to identify the psychosocial factors that are independently associated with caregiver perceptions and to highlight potential intervention targets that can support caregivers’ psychological health. This research contributes valuable insights on caregiving in pediatric oncology, with implications for developing caregiver support strategies and enhancing the overall public health response to pediatric cancer care.

## 2. Materials and methods

### 2.1 Study design and setting

This multicenter cross-sectional study was conducted from December 22, 2023 to June 15, 2024 across three hospitals affiliated with Central South University’s Xiangya School of Medicine. These hospitals are recognized for their excellence in pediatric leukemia care, clinical research, and education, while treating patients with diverse socio-economic backgrounds from all over the country. Ethical approval for the study was obtained from the institutional review board, in accordance with the Declaration of Helsinki, and written informed consent was obtained from all participants prior to data collection. Data were securely stored on password-protected systems accessible only to authorized research personnel. Participants were informed that their responses would remain strictly confidential and used solely for research purposes. They were also given the right to withdraw from the study at any time without consequences for their child’s treatment. No financial incentives were provided to avoid undue influence on participation.

### 2.2 Study population

The study targeted caregivers of pediatric patients, diagnosed with either acute lymphoblastic leukemia (ALL) or acute myeloid leukemia (AML), aged 14 or younger at the time of diagnosis, and receiving treatment at one of the three hospitals. Pediatric care in China usually refers to the care of individuals who are 14 years or younger and the standard practice for pediatric care in all three hospitals involved children in the same age range. Even though some pediatric departments may have a slightly varying age range as well as treat people older than 14 years under special circumstances, the National Cancer Center (NCC) of China reports cancer statistics following the same common practice [18]. Eligible caregivers were aged 18 or older and were actively involved in the patient’s care.

Caregivers were recruited using convenience sampling due to practical constraints, particularly the challenges of engaging caregivers who are deeply involved in the care of their children. Many pediatric oncology patients have frequent hospital visits for ongoing treatment, and a substantial number of patients are repeat visitors. Given these factors, convenience sampling allowed us to engage caregivers who were already in contact with the pediatric oncology departments, ensuring a feasible sample within the time-limited nature of the study. While convenience sampling may limit the diversity of the sample, recruiting caregivers from three hospitals helped to mitigate some potential selection bias. In addition, we distributed the questionnaires directly to caregivers who were present with their child during the study period, ensuring that the sample reflected caregivers who were actively engaged in their child’s care. Caregivers of patients with incomplete medical or treatment history, concomitant cancers, as well as those who filled less than 50% of the questionnaire were excluded.

### 2.3 Data collection

Data were collected using standardized questionnaires and medical record review. Primary variables included data from the revised Caregiving Appraisal Scale (rCAS) [19] used to evaluate both positive and negative caregiving appraisals; caregiver socio-demographic information, including age, sex, marital status, educational level, employment status, family monthly income (RMB), and their relationship to the patient; patient demographic and clinical data, such as age, sex, educational level, acute leukemia subtype, time since diagnosis, and treatment status were retrieved from the hospitals’ patient medical records. Secondary variables included residence (rural or urban), type of medical insurance, frequency of hospital visits, patient BMI class, time interval from diagnosis to treatment (days), whether or not patients received treatment elsewhere before being admitted to the current hospital, and patient symptoms—collected from caregivers—exhibited during the treatment period (anxiety about procedures, anxiety about treatment, cognitive problems, communication difficulties, nausea and vomiting, pain [5–1], worry).

#### 2.3.1 Caregiving appraisal measurement.

We adapted the rCAS, originally in English, for use in the Chinese context through a rigorous back-translation process (Brislin, 1970). First, a PhD candidate fluent in both English and Chinese translated the scale into Chinese (C-rCAS). A second post-doctoral candidate, also fluent in both languages but blind to the original version, then back-translated the C-rCAS into English. The two versions were compared to identify discrepancies, ensuring the conceptual equivalence of the C-rCAS to the original rCAS by a professor. This translation process was completed before data collection, and the Chinese version was used to ensure cultural relevance and linguistic accuracy.

The rCAS is a validated instrument that assesses caregivers’ subjective appraisal of their role across four key dimensions: caregiving mastery, satisfaction, perceived burden, and environmental impact. Positive appraisal dimensions—mastery and satisfaction—reflect caregivers’ sense of accomplishment and fulfillment, while negative appraisal dimensions—perceived burden and environmental impact—highlight emotional, physical, and social challenges. Each item on the rCAS was rated on a 5-point Likert scale, ranging from 1 (“not at all”) to 5 (“a great deal”). Domain scores were calculated by averaging the responses within each domain, yielding a score between 1 and 5 for each caregiving aspect, with higher scores indicating stronger appraisals (positive or negative depending on the dimension). The four subscales have demonstrated strong internal consistency in other publications, with Cronbach’s alpha values ranging from 0.78 to 0.89 in a study of daughters and daughters-in-law caring for elders with dementia [19]. Additionally, multiple caregiver studies, including those by Chang and Hughes & Caliandro, found that the scale maintained good internal consistency, with Cronbach’s alpha exceeding 0.70, indicating it is a reliable measure of caregiver experiences in various contexts [20,21]. We also independently validated and assessed the C-rCAS for internal consistency and reliability to ensure the trustworthiness of our results.

### 2.4 Data analysis

Statistical analyses were conducted using SPSS v27.0 (IBM Corporation, New York, USA). Descriptive statistics, such as means, standard deviations, and frequencies, were employed to summarize caregiver demographics, patient characteristics, and caregiving appraisal scores. For inferential statistics, t-tests were used to compare caregiving appraisal scores between two demographic groups (e.g., sex), and analysis of variance (ANOVA) was employed to explore differences across multiple groups (e.g., education level). Pearson’s correlation was used to investigate relationships between patient-related variables, such as discomfort, and caregiving appraisal scores.

Finally, a multiple linear regression model was developed to identify predictors of both positive and negative caregiving appraisals (self-perception). Variables found to be significant in univariate analyses (e.g., t-tests, ANOVA, and correlations) were entered into the regression model to predict caregiving outcomes, allowing for the identification of key factors independently associated with caregiving appraisal.

#### 2.4.1 Data validation, internal consistency, and reliability.

Normality of data from the C-rCAS subscales was assessed using the Kolmogorov-Smirnov and Shapiro-Wilk tests. Confirmatory factor analysis (CFA) were used to assess the validity and factor structure of the C-rCAS in this study. The Comparative Fit Index (CFI) and Cronbach’s alpha were used to assess model fit and internal consistency. The internal consistency of the C-rCAS subscales was measured using Cronbach’s alpha, with reliability for each subscale.

## 3. Results

### 3.1 Descriptive statistics

#### 3.1.1 Caregiver demographics.

**Fig 1:**
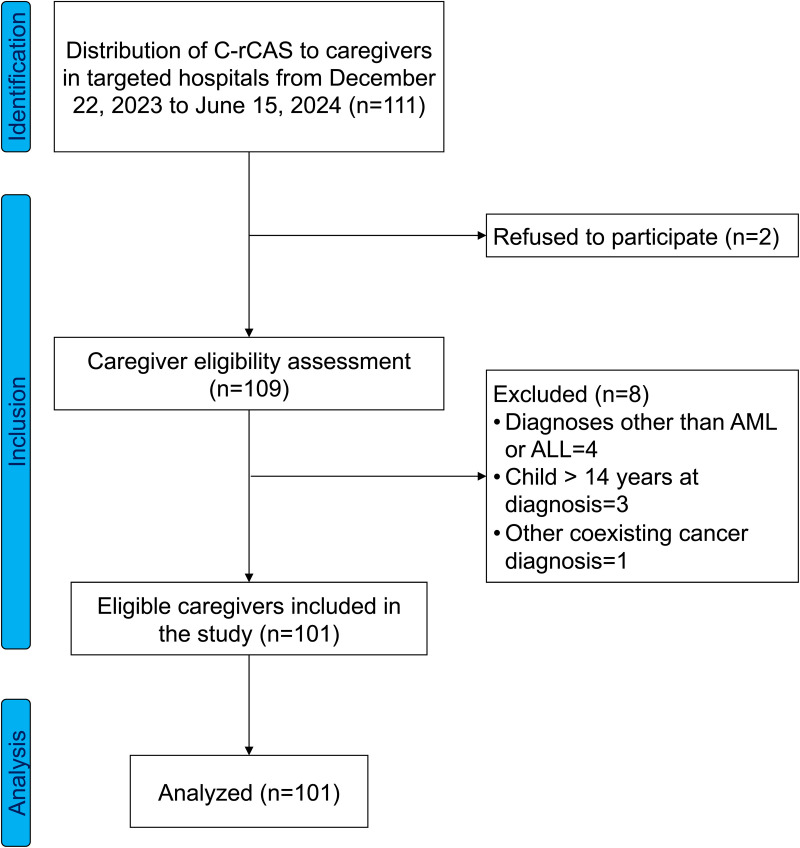
STROBE flow diagram. C-rCAS; Chinese Revised Caregiving Appraisal Scale, ALL; Acute Lymphoblastic Leukemia, AML; Acute Myeloid Leukemia.

Out of 111 caregivers approached, 2 caregivers refused to participate in the study, 4 of the 109 remaining caregivers were of children diagnosed with diseases other than acute leukemia (AL), 3 caregivers were of children whose age at the time of diagnosis was > 14 years old, and 1 caregiver was of a patient with another concomitant cancer diagnosis. Eventually, 101 valid responses were analyzed (Fig 1). The analyzed caregiver population was predominantly female (74.3%), with most caregivers aged over 35 years (68.3%). Mothers constituted the majority of caregivers (69.3%) and nearly all caregivers were married (97.1%). Educational background varied, with the largest group having completed senior high school (31.7%), followed by junior high school graduates (30.7%). Most caregivers earned less than 5,000 Chinese Yuan (RMB) per month (72.3%) and resided in rural areas (65.3%). A majority (74.3%) were covered by rural resident medical insurance. In terms of hospital visits, 59.4% of caregivers reported five or fewer hospital visits in the past six months (Table 1).

**Table 1:** Caregiver demographics.

Parameter	Frequency (n = 101)	Percent (%)
**Sex**		
**Male**	26	25.7
**Female**	75	74.3
**Age (years)**		
**26-35**	32	31.7
**> 35**	69	68.3
**Relationship to the patient**		
**Mother**	70	69.3
**Father**	26	25.7
**Extended family**	5	5.0
**Marital Status**		
**Married**	98	97.1
**Single**	3	2.9
**Educational level (Caregiver)**		
**Elementary school or below**	3	3.0
**Junior high school**	31	30.7
**Senior high school**	32	31.7
**College**	19	18.8
**Bachelor’s or higher**	16	15.8
**Family monthly income (RMB)**		
**< 5,000**	73	72.3
**5,000 - 10,000**	19	18.8
**> 10,000**	9	8.9
**Residence**		
**Rural area**	66	65.3
**Urban area**	35	34.7
**Medical insurance**		
**Commercial**	4	4.0
**Rural resident**	75	74.3
**Urban resident**	18	17.8
**Out-of-pocket**	4	4.0
**Primary caregiver**		
**Yes**	97	96.0
**No**	4	4.0
**Frequency of hospital visits in the last 6 months since diagnosis (times)**		
**≤5**	60	59.4
**6-10**	34	33.7
**>10**	7	6.9

RMB = Chinese Yuan

#### 3.1.2 Patient demographics and clinical information.

Regarding patient demographics (Table 2), 65.3% were male, and 54.5% were aged between 7 and 14 years. Most patients (79.2%) were enrolled in school, with 44.6% attending elementary school. Patient clinical data (Table 2) showed that the most common diagnosis was B-ALL (66.3%). Most patients (74.3%) started chemotherapy within three days of diagnosis, with more than half (53.5%) having undergone treatment for six months or less. At the time of the study, 38.6% were in the induction phase of treatment.

**Table 2:** a. Patient demographics. b. Patients’ clinical information.

Parameter	Frequency (n = 101)	Percent (%)
a. Patient demographics		
**Sex**		
**Male**	66	65.3
**Female**	35	34.7
**Age (year)**		
**1-3**	17	16.8
**4-6**	23	22.8
**7-14**	55	54.5
**>14**	6	5.9
**Education**		
**Yes**	80	79.2
**No**	21	20.8
**Educational level**		
**Preschool or below**	19	18.8
**Elementary school**	45	44.6
**Middle school**	13	12.9
**High school**	3	3.0
		
b. Patients’ clinical information		
**Classification of acute leukemia**		
**B-ALL**	67	66.3
**T-ALL**	16	15.8
**AML**	18	17.8
**Time since diagnosis**		
**≤1 month**	23	22.8
**1-6 months**	30	29.7
**6 months-1 year**	24	23.8
**1-5 years**	24	23.8
**BMI classification**		
**Underweight**	8	7.9
**Normal**	77	76.2
**Overweight**	10	9.9
**Obese**	6	5.9
**Total hospital visits after diagnosis (times)**		
**<5**	49	48.5
**5-10**	25	24.8
**>10**	27	26.7
**Days interval from diagnosis to starting chemotherapy**		
**≤3**	75	74.3
**4-7**	18	17.8
**>7**	8	7.9
**Duration of chemotherapy (months)**		
**≤6**	54	53.5
**>6-12**	23	22.8
**>12-24**	15	14.9
**>24**	9	8.9
**Current stage of the latest chemotherapy**		
**Induction**	39	38.6
**Consolidation**	18	17.8
**Intensification or maintenance**	44	43.6
**Pre-admission treatment**		
**Yes**	36	35.6
**No**	65	64.4

B-ALL = B-cell Acute Lymphocytic Leukemia, T-ALL = T-cell Acute Lymphocytic Leukemia, AML = Acute Myelocytic Leukemia, BMI = Body Mass Index

### 3.2 Test of normality

Kolmogorov-Smirnov and Shapiro-Wilk tests indicated normal distribution for all key variables: burden, satisfaction, mastery, and environment (Table 3).

**Table 3:** Normality test.

	Kolmogorov-Smirnova			Shapiro-Wilk		
	Statistic	df	Sig.	Statistic	df	Sig.
**Burden**	0.078	101	0.14	0.983	101	0.208
**Satisfaction**	0.12	101	0.177	0.951	101	0.141
**Mastery**	0.12	101	0.307	0.968	101	0.192
**Environment**	0.121	101	0.291	0.949	101	0.219

Sig.; significance, df; degrees of freedom

### 3.3 Reliability and validity analyses

Reliability and validity analysis (Table 4) demonstrated that the Burden subscale, consisting of nine items, had strong internal consistency (Cronbach’s α = 0.818, 95% CI: [0.782, 0.945]). The Satisfaction subscale with six items showed acceptable internal consistency (Cronbach’s α = 0.691, 95% CI: [0.523, 0.767]). The Mastery subscale, comprising four items, showed low internal consistency (Cronbach’s α = 0.542, 95% CI: [0.439, 0.624]), and the Environment subscale (three items) exhibited good reliability (Cronbach’s α = 0.767, 95% CI: [0.641, 0.862]). The overall model fit was evaluated using the Comparative Fit Index (CFI = 0.764), indicating an adequate fit of the scale structure to the data.

**Table 4:** Reliability and validity analysis.

				95% CI			
	No. of Items	N	Cronbach’s Alpha	Lower	Upper	Eigen value	CFI
**Burden**	9	101	0.818	0.782	0.945	6.509	0.764
**Satisfaction**	6	101	0.691	0.523	0.767	5.281	
**Mastery**	4	101	0.542	0.439	0.624	2.744	
**Environment**	3	101	0.767	0.641	0.862	2.047	

N = sample number, CFI = Comparative Fit Index

### 3.4 Univariate analysis

#### 3.4.1 Caregiver-related factors influencing positive and negative appraisals.

Caregiver satisfaction was influenced by the relationship to the patient, family income, and medical insurance type. Extended family members *(x̄* = 35.1, *F* = 4.395, *P* = 0.004) reported higher satisfaction while caregivers earning 5,000–10,000 RMB (*x̄* = 21.7, *F* = 3.162, *P* = 0.017), and urban resident insurance holders *(x̄* = 20.8, F = 4.479, *P* = 0.005) reported the lowest satisfaction in their respective subgroups.

Mastery was influenced by age, relationship to the patient, marital status, educational level, income, medical insurance, and frequency of hospital visits. Caregivers aged > 35 years (*x̄* = 15.4, *t* = 3.180, *P* = 0.041), ex*t*ended family members (*x̄* = 17.3, *F* = 5.896, p < 0.001), and unmarried caregivers (*x̄* = 16.0, *F* = 3.154, *P* = 0.047) reported the highest mastery appraisals; elementary school graduates *(x̄* = 15.3) and junior high graduates (*x̄* = 15.6) reported significantly higher mastery compared to college (*x̄* = 13.3) and bachelor’s degree holders (*x̄* = 12.3) (*F* = 3.078, *P* = 0.041); Caregivers with family income > 10,000 RMB (*x̄* = 11.9, *F* = 2.054, *P* = 0.042), urban resident insurance holders (*x̄* = 12.3, *F* = 3.250, *P* = 0.025), and caregivers of children with > 10 hospital visits in the last six months *(x̄* = 12.0, *F* = 2.660, *P* = 0.035) reported significantly lowest mastery appraisals in their respective subgroups (S1 Table).

Caregiver burden varied significantly based on the caregiver’s relationship to the patient, education level, family income, medical insurance type, and whether they were the primary caregiver. The following subgroups of caregivers reported significantly higher burden relative to their other respective subgroups. Extended family members (*x̄* = 35.1, *F* = 5.428, *P* < 0.001); Caregivers with the lowest educational background, elementary school graduates *(x̄* = 34.7, *F* = 4.203, *P* = 0.004); Caregivers with family income < 5,000 RMB (*x̄* = 26.6, *F* = 3.746, *P* = 0.027); Commercial insurance holders reported the highest burden (*x̄* = 28.3) while urban insurance holders reported significantly lower burden (*x̄* = 22.2) (*F* = 3.788, *P* = 0.015); Primary caregivers also reported higher burden (*x̄* = 25.7, *t* = 5.080, *P* = 0.028).

Environmental impact was significantly influenced by the caregiver’s relationship to the patient and marital status. Extended family members experienced higher environmental impact (*x̄* = 10.0, *F* = 2.599, *P* = 0.017); Unmarried caregivers (including single and divorced caregivers) reported higher environmental impact (*x̄* = 12.8, *t* = 3.573, *P* = 0.032) (S2 Table).

#### 3.4.2 Patient-related factors influencing positive and negative appraisals.

For satisfaction, caregivers of patients undergoing chemotherapy for more than 24 months reported the highest satisfaction (*x̄* = 26.3, *F* = 5.392, *P* = 0.002). No patient factors were associated with variance in caregiver mastery (S3 Table).

Time since diagnosis, total hospital visits, and the time interval from diagnosis to chemotherapy initiation were significant factors affecting caregiver burden. Caregivers of patients diagnosed in the past six months at the time of filling the questionnaire reported significantly lower burden *(x̄* = 24.8, *F* = 3.553, *P* = 0.047); Burden was higher for caregivers with more than ten hospital visits (*x̄* = 27.2, *F* = 4.814, *P* = 0.016); Caregivers of patients who initiated chemotherapy within 3 days of diagnosis reported lower burden (*x̄* = 21.5, *F* = 6.545, *P* = 0.002).

Regarding environmental impact, caregivers of patients who started chemotherapy within 4–7 days of diagnosis reported significantly higher environmental burden (*x̄* = 10.6, *F* = 3.917, *P* = 0.023). The duration of chemotherapy also influenced environmental impact, with caregivers of patients undergoing chemotherapy for 6–12 months reporting the highest environmental (*x̄* = 27.5, *F* = 4.469, *P* = 0.036) (S4 Table).

### 3.5 Correlation analysis

Significant correlations were observed between patient discomfort and caregiver appraisals. Burden correlated positively with patient worry (*r* = 0.237, *P* < 0.05) and communication difficulties (*r* = 0.210, *P* < 0.05). Satisfaction was negatively correlated with patient pain (*r* = -0.334, *P* < 0.001) and anxiety about surgical procedures (*r* = -0.296, *P* < 0.001). Mastery was negatively correlated with patient pain (*r* = -0.210, *P* < 0.05) and anxiety about surgical procedures (*r* = -0.212, *P* < 0.05). Environmental impact was positively correlated with patient anxiety about surgical procedures (*r* = 0.259, *P* < 0.001) and anxiety about treatment (*r* = 0.333, *P* < 0.001) (S5 Table).

### 3.6 Multivariate regression analysis

#### 3.6.1 Positive appraisal regression model.

The model for positive caregiving appraisal was also statistically significant (*F* = 4.352, *P* < 0.001), accounting for 30.3% of the variance (R² = 0.303). Significant predictors included pain (*β* = -1.937, 95% CI [-3.27, -0.446], *P* = 0.008), anxiety about surgical procedures (*β* = -2.757, 95% CI [-3.831, -1.482], *P* = 0.022), relationship to the patient (*β* = 1.94, 95% CI [0.084, 2.966], *P* = 0.031), and caregiver educational level (*β* = -1.437, 95% CI [-2.696, -0.227], *P* = 0.023) (Table 5).

**Table 5:** Linear regression model of significant factors affecting positive caregiving appraisal.

	Unstandardized Coefficients		Standardized Coefficients	*t*	Sig.	95% CI	
	*β*	Std. Error	*β*			Lower	Upper
**(Constant)**	51.71	5.357		9.653	0	41.856	62.774
**Age (years)**	-1.56	1.191	-0.122	-1.31	0.194	-2.943	0.804
**Anxiety about medical procedures**	-2.757	0.586	-0.134	-1.292	**0.022**	-3.831	-1.482
**Duration of chemotherapy**	0.744	0.557	0.126	1.335	0.185	-0.037	0.16
**Educational level (Caregiver)**	-1.437	0.62	-0.271	-2.319	**0.023**	-2.696	-0.227
**Family monthly income (RMB)**	1.23	1.07	0.134	1.15	0.253	0.917	2.339
**Marital Status**	-0.329	3.191	-0.067	-0.73	0.146	-0.824	0.132
**Medical insurance**	-1.611	0.961	-0.157	-1.676	0.097	-2.848	0.271
**Pain [5–1]**	-1.937	0.714	-0.28	-2.714	**0.008**	-3.27	-0.446
**Relationship to the patient**	1.94	0.979	0.189	1.981	**0.031**	0.084	2.966

Dependent Variable: positive caregiving appraisal, *F *= 4.352, *P* < .001, R^2^ = 0.303, *β* = Beta, *t* = *t*-statistic, Sig. = Significance, CI = Confidence Interval

#### 3.6.2 Negative appraisal regression model.

The regression model for negative caregiving appraisal was statistically significant (*F* = 2.067, *P* = 0.024), explaining 23.9% of the variance (R² = 0.239). Significant predictors included patient worry (*β* = 0.379, 95% CI [0.056, 2.446], *P* = 0.024), communication difficulties (*β* = 1.204, 95% CI [0.573, 2.977], *P* = 0.011), relationship to the patient (*β* = 2.921, 95% CI [0.535, 5.378], *P* = 0.017), and medical insurance type (*β* = -1.853, 95% CI [-3.094, -0.387], *P* = 0.035) (Table 6).

**Table 6:** Linear regression model of significant factors affecting negative caregiving appraisal.

	Unstandardized Coefficients		Standardized Coefficients	*t*	Sig.	95% CI	
	*β*	Std. Error	*β*			Lower	Upper
**(Constant)**	37.209	8.541		4.356	0	20.23	54.189
**Anxiety about surgical procedures**	1.305	1.185	0.149	1.101	0.274	-1.051	2.662
**Anxiety about treatment**	0.103	1.224	0.013	0.084	0.933	-2.329	2.536
**Communication difficulties**	1.204	0.892	0.144	3.349	**0.011**	0.573	2.977
**Days interval from diagnosis to starting chemo**	-0.341	0.293	-0.12	-1.163	0.248	-0.923	0.242
**Educational level (Caregiver)**	-1.808	1.049	-0.22	-1.723	0.088	-3.894	0.278
**Family monthly income (RMB)**	-0.048	1.794	-0.003	-0.027	0.979	-1.615	1.519
**Marital Status**	4.504	5.268	0.084	0.855	0.395	-5.969	14.976
**Medical insurance**	-1.853	1.63	-0.117	-2.137	**0.035**	-3.094	-0.387
**Primary caregiver**	-0.629	0.926	-0.163	-1.549	0.125	-1.742	2.163
**Relationship to the patient**	2.921	1.739	0.183	3.681	**0.017**	0.535	5.378
**Time since diagnosis (months)**	-0.134	0.093	-0.162	-1.437	0.154	-0.319	0.051
**Total hospital visits after diagnosis**	0.045	0.124	0.041	0.362	0.718	-0.202	0.292
**Worry**	0.379	1.044	0.047	2.354	**0.024**	0.056	2.446

Dependent Variable: negative caregiving appraisal, *F* = 2.067, *P* = 0.024, R^2^ = 0.239, *β* = Beta, *t* = t-statistic, Sig. = Significance, CI = Confidence Interval

## 4. Discussion

The findings from this study offer significant insights into the complex psychological and socio-economic challenges faced by caregivers of pediatric acute leukemia patients. Caregivers, particularly parents and extended family members, bear an immense emotional, financial, and environmental burden, which is exacerbated by various patient-related factors, such as the child’s treatment duration, physical discomfort, and communication difficulties. These findings emphasize the need for targeted interventions and support systems for caregivers, especially those with lower socio-economic status and limited access to healthcare resources.

Majority of the caregivers were female (74.3%), aligning with global trends showing that caregiving responsibilities disproportionately fall on women most likely due to traditional gender roles within families [22,23]. Mothers (69.3%) were the predominant caregivers, which may be attributed to their central role in family caregiving across various cultures. This uneven gender distribution among caregivers is consistent with studies that demonstrate the critical role of mothers in managing the emotional and physical needs of children with chronic illnesses [24]. The age distribution of caregivers, with 68.3% aged over 35 years may reflect a broader social trend where families today have children later in life. In the same vein, older individuals are likely to have more established family structures and potentially greater life stability but may also face age-related challenges such as physical fatigue and mental strain. These challenges are particularly relevant when caring for patients who require long-term treatment as older caregivers have been shown to experience higher levels of emotional distress, which can exacerbate the psychological toll of caregiving [25,26]. This reinforces the need for specific support strategies that address the age-related challenges caregivers face, especially as they balance caregiving with their own health concerns.

The psychological strain experienced by caregivers was apparent in the reported burden and environmental impact. Extended family members reported significantly higher burden scores compared to mothers and fathers. This result aligns with existing literature suggesting that non-primary caregivers, particularly extended family members, may feel less prepared for the caregiving role and consequently experience higher levels of stress and burden [8,27]. Extended family members often step into caregiving roles under urgent circumstances, without the same emotional investment or prior experience that primary caregivers may have, leading to increased feelings of helplessness and lack of control over caregiving tasks. Caregivers who had lower educational backgrounds reported higher levels of burden. This trend supports previous findings that indicate individuals with limited education often face difficulties understanding complex medical information, managing healthcare tasks, and accessing appropriate resources, which exacerbates stress [17]. The data suggests that caregivers with elementary education experience the most significant burden, while those with higher education levels report a reduced burden, likely due to greater familiarity with healthcare systems and increased capacity to advocate for their children’s needs.

Lower-income families experienced the highest levels of burden. This finding underscores the financial strain placed on families managing chronic illnesses such as pediatric leukemia. The lower a family’s income, the more likely they are to face considerable challenges in affording medical care, transportation, and daily living expenses, further intensifying their caregiving burden [28,29]. Caregivers with commercial insurance reported significantly higher levels of burden, possibly reflecting the out-of-pocket costs often associated with such plans. This result contrasts with the burden experienced by rural residents, who may rely on rural insurance schemes that offer limited coverage [30]. Unmarried caregivers reported higher environmental stress compared to their married counterparts. The lack of spousal support may explain this increased burden, as single caregivers are left to manage caregiving responsibilities without the emotional and practical support that married individuals often have. This finding aligns with other studies that emphasize the protective effect of marital status on caregiving outcomes, as married caregivers generally have better psychological resilience due to shared caregiving responsibilities [31].

Higher mastery was reported by caregivers with lower education and income levels, which initially appears counterintuitive. A person’s level of education plays a role in access to resources and knowledge, and caregivers with higher education levels generally have better access to healthcare resources and information, which can alleviate caregiving challenges [32]. However, caregivers without these resources are more likely to take on full caregiving responsibilities, which can enhance their sense of mastery. Nevertheless, this increased involvement is positively associated with greater caregiver burden. In the present study, the coexistence of higher perceived burden and higher perceived mastery was observed. Caregivers reported experiencing significant emotional and psychological strain, which is often associated with feelings of burden. However, they also reported a sense of mastery and satisfaction. This may reflect a psychological adaptation process, where caregivers in more vulnerable situations develop greater resilience or derive fulfillment from overcoming daily challenges. A study involving Chinese pediatric leukemia parent caregivers noted that caregivers seem to go through several stages of psychologically adapting to their role (from initial devastation to eventual adaptation), a process which was accompanied with different emotions [33]. At the same time, caregivers who face more difficult circumstances often develop greater resilience and problem-solving abilities, leading to a sense of mastery. For example, caregivers who have been involved in the caregiving process for longer periods may feel they have become more competent in managing the daily demands of care. This mastery can provide a sense of accomplishment, despite the significant burden. The dual experience of burden and mastery highlights the complexity of caregiving, where emotional and psychological strain is often intertwined with feelings of empowerment and resilience [34]. There is evidence that perceived burden often reported by caregivers can be mitigated by social support [35].

Regarding income, parents with fewer financial resources tend to assume more caregiving responsibility, which could contribute to greater perceived competence. However, this is often accompanied by a greater caregiving burden, as they lack the support systems and resources that could alleviate the strain. The aforementioned dual burden-mastery experience is exhibited here too. These dynamics suggest that caregivers in financially constrained situations may develop greater resilience, but without proper support, the burden becomes overwhelming. As such, there is a need for healthcare systems to apply targeted interventions that not only focus on reducing caregiver burden by more subsidized treatment options but also leverage caregivers’ inherent strengths and support them in ways that improve upon their resilience by providing training that builds their skills and confidence in managing care as well as encouraging them to identify and interact with supportive friends and family.

Although longer treatment times often correlate with increased caregiver burden due to frequent hospital visits, caregivers of children undergoing longer treatment durations reported higher satisfaction. This may reflect their ability to see progress and improvement in the child’s health over time. Also, caregivers often express that providing long term care brings a sense of personal fulfillment, making them feel valued and important. Moreover, the satisfaction reported by caregivers could stem from the belief that their prolonged involvement is making a tangible difference in the child’s health and quality of life. Lastly, long-term care often creates stronger emotional bonds between caregivers and patients, further contributing to feelings of personal satisfaction and emotional reward [8,36–38]. This finding suggests that while longer treatment leads to higher caregiver burden, it can also foster a sense of personal accomplishment and meaning. Therefore, interventions aimed at improving caregiver well-being should be longitudinal, supporting caregivers throughout the entirety of the pediatric patient’s treatment trajectory. Such approaches should be integrated into global pediatric oncology care frameworks, particularly in regions where long-term support services are scarce.

Patient-related factors such as the time since diagnosis, total hospital visits, and the interval between diagnosis and chemotherapy initiation were significant predictors of caregiver burden. Caregivers of patients who were newly diagnosed (within 1–6 months) reported lower levels of burden compared to those whose children had been living with the disease for longer. This finding is consistent with prior research suggesting that the initial period after diagnosis is marked by acute emotional distress, which transitions into chronic strain as caregivers adjust to the long-term demands of caregiving [8].

Similarly, the frequency of hospital visits and the duration of chemotherapy were key factors influencing caregiver burden. Caregivers of children who required more frequent hospital visits and those undergoing prolonged chemotherapy reported significantly higher levels of burden. This is likely due to the cumulative effect of frequent healthcare interactions, which disrupt daily life and contribute to a sense of isolation and emotional exhaustion.

The environmental impact on caregivers, including disruptions to work and social life, was notably higher among unmarried caregivers and those facing frequent hospital visits. Unmarried caregivers may lack the social support network available to married individuals, compounding the strain of caregiving responsibilities [31]. Additionally, the necessity of frequent hospital visits, particularly for families in rural areas, imposes logistical and financial challenges that further exacerbate environmental stress.

A critical finding in this study was the significant correlation between the child’s physical discomfort and the caregiving appraisals. Higher levels of worry, communication difficulties, and anxiety about medical procedures in the child were associated with greater caregiver burden, lower satisfaction, and decreased feelings of mastery. This suggests that the emotional and physical well-being of the child plays a direct role in shaping the caregiver’s psychological state. In line with this, younger children in the study were found to have worse treatment experiences, particularly marked by elevated levels of procedural anxiety. These issues were more prominent among the younger patients, reflecting the unique challenges they face during treatment, which in turn exacerbates caregiver stress [39].

Mothers with lower educational levels, who may have an incomplete understanding of their child’s diagnosis, are especially prone to overprotective behaviors due to emotional sensitivity and protective instincts. Such behaviors can amplify the child’s experiences of pain, anxiety, and communication difficulties, further intensifying the psychological toll on both the child and the caregiver. This dynamic between maternal caregiving behaviors and child well-being, contributing to increased caregiver burden has been highlighted in previous research [40].

These correlations reinforce the importance of addressing not only the physical health of the child but also the psychological well-being of the caregiver. Interventions aimed at reducing patient discomfort, particularly pain management and anxiety reduction during medical procedures, could significantly improve caregiving appraisals and in turn, patient outcomes. There is need for a more holistic approach to pediatric leukemia care, one that includes mental health support for caregivers alongside medical treatment for the patient.

The findings from this study underline the urgent need for targeted interventions to support caregivers of children with PAL. Given the significant burden faced by caregivers with lower socio-economic status, financial assistance programs and improved access to comprehensive healthcare coverage could alleviate some of the strain associated with caregiving. Moreover, educational programs tailored to caregivers with lower levels of formal education may empower them with the knowledge and skills necessary to manage complex medical situations more effectively.

Additionally, psychological support services, such as counseling and support groups, should be made readily available to caregivers, particularly those dealing with high levels of patient-related anxiety and discomfort. These services can provide caregivers with coping strategies to manage the emotional challenges of caregiving, ultimately improving their mental health and ability to provide care.

A key strength of this study is its comprehensive assessment of both caregiver-related and patient-related factors influencing caregiver burden, satisfaction, mastery, and environmental impact. However, the study also has limitations. The cross-sectional design limits the ability to establish causal relationships, and the self-reported nature of the data as well as the convenience sampling method used to recruit participants may introduce bias. Future studies should consider a longitudinal design to better capture the dynamic changes in caregiver experiences over time.

## 5. Conclusion

This study identifies the significant psychological burden and environmental impact faced by caregivers of pediatric acute leukemia patients while also highlighting the presence of positive caregiving appraisals such as satisfaction and mastery, all associated with various patient- and caregiver-related factors. Interventions that reduce caregiver burden and enhance caregiving confidence, especially for vulnerable caregiver subgroups, are essential for improving the psychological well-being of caregivers.

## Supporting information

S1 Table Caregiver factors influencing positive caregiving appraisal. *x̄*; mean, *t*; t-test statistic for two groups, *F*; F-test statistic for more than two groups, *P*-value ≤ 0.05 indicates statistical significance.
(DOCX)

S2 Table Caregiver factors influencing negative caregiving appraisal. *x̄*; mean, *t*; t-test statistic for two groups, *F*; F-test statistic for more than two groups, *P*-value ≤ 0.05 indicates statistical significance.
(DOCX)

S3 Table Patient factors influencing positive caregiving appraisal. *x̄*; mean, *t*; t-test statistic for two groups, *F*; F-test statistic for more than two groups, *P*-value ≤ 0.05 indicates statistical significance.
(DOCX)

S4 Table Patient factors influencing negative caregiving appraisal. *x̄*; mean, *t*; t-test statistic for two groups, *F*; F-test statistic for more than two groups, *P*-value ≤ 0.05 indicates statistical significance.
(DOCX)

S5 Table Correlation matrix of patient symptoms and caregiving appraisal. *; p < .05, **; p < .001.
(DOCX)
