# Supplementary material for: A multicenter cross-sectional study on factors associated with caregiving appraisal in pediatric acute leukemia caregivers
Source: PLoS One. 2025 Jun 6;20(6):e0324589. doi: 10.1371/journal.pone.0324589 (PMC12143579; doi:10.1371/journal.pone.0324589)
Supplement: S1 Table — x̄; mean, t; t-test statistic for two groups, F; F-test statistic for more than two groups, P-value ≤ 0.05 indicates statistical significance. (DOCX) [file pone.0324589.s001.docx]

**S1 Table. Caregiver Factors Influencing Positive Caregiving Appraisal**

|  | Satisfaction | | | Mastery | | |
| --- | --- | --- | --- | --- | --- | --- |
|  | *x̄* | *t/F* | *P*-value | Mean *x̄* | *t/F* | *P*-value |
| Sex |  |  |  |  |  |  |
| Male | 24.5 | 0.920 | 0.360 | 14.2 | 0.270 | 0.788 |
| Female | 23.6 |  |  | 14.1 |  |  |
| Age (years) |  |  |  |  |  |  |
| 26-35 | 24.2 | 0.594 | 0.554 | 13.6 | 3.180 | **0.041** |
| > 35 | 23.7 |  |  | 15.4 |  |  |
| Relationship to the patient | |  |  |  |  |  |
| Mother | 25.4 | 4.395 | **0.004** | 13.7 | 5.896 | **0.001** |
| Father | 23.7 |  |  | 14.2 |  |  |
| Extended family | 35.1 |  |  | 17.3 |  |  |
| Marital Status |  |  |  |  |  |  |
| Married | 23.8 | 1.094 | 0.065 | 14.0 | 3.154 | **0.047** |
| Single | 23.0 |  |  | 16.0 |  |  |
| Educational level | |  |  |  |  |  |
| Elementary | 24.7 | 0.977 | 0.424 | 15.3 | 3.078 | **0.041** |
| Junior high | 24.3 |  |  | 15.6 |  |  |
| Senior high | 24.5 |  |  | 13.9 |  |  |
| College | 23.3 |  |  | 13.3 |  |  |
| Bachelor's | 22.3 |  |  | 12.3 |  |  |
| Family monthly income (RMB) | |  |  |  |  |  |
| < 5,000 | 26.6 | 3.162 | **0.017** | 14.3 | 2.054 | **0.042** |
| 5,000-10,000 | 21.7 |  |  | 14.2 |  |  |
| > 10,000 | 24.8 |  |  | 11.9 |  |  |
| Residence |  |  |  |  |  |  |
| Rural area | 23.9 | 0.109 | 0.913 | 14.5 | 1.875 | 0.064 |
| Urban area | 23.8 |  |  | 13.4 |  |  |
| Medical insurance |  |  |  |  |  |  |
| Commercial | 24.5 | 4.479 | **0.005** | 15.0 | 3.250 | **0.025** |
| Rural resident | 24.5 |  |  | 14.5 |  |  |
| Urban resident | 20.8 |  |  | 12.3 |  |  |
| Out-of-pocket | 25.5 |  |  | 13.3 |  |  |
| Primary caregiver |  |  |  |  |  |  |
| Yes | 23.9 | 0.055 | 0.956 | 14.1 | -0.107 | 0.915 |
| No | 23.8 |  |  | 14.2 |  |  |
| Frequency of hospital visits in the last 6 months since diagnosis (times) | | | | |  |  |
| ≤5 | 23.5 | 1.210 | 0.303 | 14.0 | 2.660 | **0.035** |
| 6-10 | 24.7 |  |  | 14.7 |  |  |
| >10 | 22.7 |  |  | 12.0 |  |  |

*x̄*; mean, *t*; t-test statistic for two groups, *F*; F-test statistic for more than two groups, *P*-value ≤ 0.05 indicates statistical significance
