# Supplementary material for: A multicenter cross-sectional study on factors associated with caregiving appraisal in pediatric acute leukemia caregivers
Source: PLoS One. 2025 Jun 6;20(6):e0324589. doi: 10.1371/journal.pone.0324589 (PMC12143579; doi:10.1371/journal.pone.0324589)
Supplement: S2 Table — x̄; mean, t; t-test statistic for two groups, F; F-test statistic for more than two groups, P-value ≤ 0.05 indicates statistical significance. (DOCX) [file pone.0324589.s002.docx]

**S2 Table. Caregiver Factors Influencing Negative Caregiving Appraisal**

|  | Burden | | | | Environment | | | | | |  |  |  |
| --- | --- | --- | --- | --- | --- | --- | --- | --- | --- | --- | --- | --- | --- |
| Parameter | *x̄* | *t/F* | *P*-value | *x̄* | | *t/F* | | *P*-value | | |  |  |  |
| Sex |  |  |  |  | | |  | |  | | | | |
| Male | 24.6 | -0.778 | 0.439 | 7.5 | | | -0.505 | | 0.615 | | |  |  |
| Female | 25.8 |  |  | 7.9 | | |  | |  | | |  |  |
| Age (years) |  |  |  |  | | |  | |  | | |  |  |
| 26-35 | 25.3 | -0.193 | 0.847 | 7.5 | | | -0.584 | | 0.561 | | |  |  |
| > 35 | 25.6 |  |  | 7.9 | | |  | |  | | |  |  |
| Relationship to the patient |  |  |  |  | | |  | | | | | |  |
| Mother | 25 | 5.428 | **0.002** | 7.7 | | | 2.599 | | **0.017** | | |  |  |
| Father | 24.6 |  |  | 7.5 | | |  | |  | | |  |  |
| Extended family | 35.1 |  |  | 10 | | |  | |  | | |  |  |
| Marital Status |  |  |  |  | | |  | |  | | |  |  |
| Married | 25.4 | 0.668 | 0.515 | 7.6 | | | 3.573 | | **0.032** | | |  |  |
| Single | 30.5 |  |  | 12.8 | | |  | |  | | |  |  |
| Educational level |  |  |  |  | | |  | | | | | |  |
| Elementary | 34.7 | 4.203 | **0.004** | 9 | | | 0.345 | | 0.847 | | |  |  |
| Junior high | 28.4 |  |  | 7.5 | | |  | |  | | |  |  |
| Senior high | 24.7 |  |  | 8.1 | | |  | |  | | |  |  |
| College | 22.6 |  |  | 7.4 | | |  | |  | | |  |  |
| Bachelor’s | 23.4 |  |  | 7.9 | | |  | |  | | |  |  |
| Family monthly income (RMB) |  |  |  |  | | |  | | | | | |  |
| < 5,000 | 26.6 | 3.746 | **0.027** | 7.9 | | | 2.263 | | 0.109 | | |  |  |
| 5,000 - 10000 | 21.7 |  |  | 6.6 | | |  | |  | | |  |  |
| > 10000 | 24.8 |  |  | 9.2 | | |  | |  | | |  |  |
| Residence |  |  |  |  | | |  | |  | | |  |  |
| Rural area | 26.3 | 1.598 | 0.113 | 7.8 | | | 0.198 | | 0.843 | | |  |  |
| Urban area | 24 |  |  | 7.7 | | |  | |  | | |  |  |
| Medical insurance |  |  |  |  | | |  | |  | | |  |  |
| Commercial | 28.3 | 3.788 | **0.015** | 10 | | | 0.833 | | 0.479 | | |  |  |
| Rural resident | 26.2 |  |  | 7.7 | | |  | |  | | |  |  |
| Urban resident | 22.2 |  |  | 7.9 | | |  | |  | | |  |  |
| Out-of-pocket | 25.3 |  |  | 6.8 | | |  | |  | | |  |  |
| Primary caregiver |  |  |  |  | | |  | |  | | | | |
| Yes | 25.7 | 5.08 | **0.028** | 7.9 | | | 1.466 | | 0.146 |  |  |  |  |
| No | 21.8 |  |  | 5.5 | | |  | |  |  |  |  |  |
| Frequency of hospital visits in the last 6 months since diagnosis (times) |  |  |  |  |  |  |  |  |  |  |  |  |  |
| ≤5 | 25.3 | 0.148 | 0.863 | 7.7 | | | 0.076 | | 0.927 |  |  |  |  |
| 6-10 | 26.1 |  |  | 7.9 | | |  | |  |  |  |  |  |
| >10 | 25.1 |  |  | 7.6 | | |  | |  |  |  |  |  |

*x̄*; mean, *t*; t-test statistic for two groups, *F*; F-test statistic for more than two groups, *P*-value ≤ 0.05 indicates statistical significance
