# Supplementary material for: A multicenter cross-sectional study on factors associated with caregiving appraisal in pediatric acute leukemia caregivers
Source: PLoS One. 2025 Jun 6;20(6):e0324589. doi: 10.1371/journal.pone.0324589 (PMC12143579; doi:10.1371/journal.pone.0324589)
Supplement: S3 Table — x̄; mean, t; t-test statistic for two groups, F; F-test statistic for more than two groups, P-value ≤ 0.05 indicates statistical significance. (DOCX) [file pone.0324589.s003.docx]

**S3 Table. Patient Factors Influencing Positive Caregiving Appraisal**

|  | Satisfaction | | | Mastery | | |
| --- | --- | --- | --- | --- | --- | --- |
|  | *x̄* | *t/F* | *P*-value | *x̄* | *t/F* | *P*-value |
| Classification of acute leukemia | |  |  |  |  |  |
| B-ALL | 23.5 | 1.104 | 0.336 | 14.0 | 0.072 | 0.931 |
| T-ALL | 25.1 |  |  | 14.1 |  |  |
| AML | 24.2 |  |  | 14.3 |  |  |
| Time since diagnosis | |  |  |  |  |  |
| ≤1 month | 23.8 | 0.078 | 0.972 | 14.3 | 0.226 | 0.878 |
| 1-6 months | 23.8 |  |  | 13.8 |  |  |
| 6 months-1 year | 23.7 |  |  | 14.1 |  |  |
| 1-5 years | 24.2 |  |  | 14.4 |  |  |
| BMI classification |  |  |  |  |  |  |
| Underweight | 24.9 | 0.213 | 0.887 | 14.8 | 0.165 | 0.920 |
| Normal | 23.7 |  |  | 14.0 |  |  |
| Overweight | 24.2 |  |  | 14.2 |  |  |
| Obese | 23.8 |  |  | 13.8 |  |  |
| Total hospital visits after diagnosis (times) | | |  |  |  |  |
| <5 | 23.7 | 0.078 | 0.925 | 13.8 | 0.678 | 0.105 |
| 5-10 | 24.0 |  |  | 14.4 |  |  |
| >10 | 24.0 |  |  | 14.4 |  |  |
| Days interval from diagnosis to starting chemotherapy | | | |  |  |  |
| ≤3 | 23.7 | 1.722 | 0.068 | 14.0 | 0.116 | 0.891 |
| 4-7 | 23.9 |  |  | 14.2 |  |  |
| >7 | 22.5 |  |  | 14.5 |  |  |
| Duration of chemotherapy (months) | |  |  |  |  |  |
| ≤6 months | 23.8 | 5.392 | **0.002** | 13.9 | 1.438 | 0.076 |
| >6-12 months | 23.6 |  |  | 14.1 |  |  |
| >12-24 months | 22.9 |  |  | 13.9 |  |  |
| >24 months | 26.3 |  |  | 15.1 |  |  |
| Current stage of the latest chemotherapy | |  |  |  |  |  |
| Induction | 23.5 | 0.651 | 0.224 | 13.7 | 0.553 | 0.577 |
| Consolidation | 24.8 |  |  | 14.3 |  |  |
| Intensification or maintenance | 23.8 |  |  | 14.3 |  |  |
| Pre-admission treatment |  |  |  |  |  |  |
| Yes | 23.8 | -0.152 | 0.880 | 14.1 | 0.103 | 0.918 |
| No | 23.9 |  |  | 14.1 |  |  |

*x̄*; mean, *t*; t-test statistic for two groups, *F*; F-test statistic for more than two groups, *P*-value ≤ 0.05 indicates statistical significance
