# Supplementary material for: A multicenter cross-sectional study on factors associated with caregiving appraisal in pediatric acute leukemia caregivers
Source: PLoS One. 2025 Jun 6;20(6):e0324589. doi: 10.1371/journal.pone.0324589 (PMC12143579; doi:10.1371/journal.pone.0324589)
Supplement: S4 Table — x̄; mean, t; t-test statistic for two groups, F; F-test statistic for more than two groups, P-value ≤ 0.05 indicates statistical significance. (DOCX) [file pone.0324589.s004.docx]

**S4 Table. Patient Factors Influencing Negative Caregiving Appraisal**

|  | Burden | | | Environment | | |
| --- | --- | --- | --- | --- | --- | --- |
|  | *x̄* | *t/F* | *P*-value | *x̄* | *t/F* | *P*-value |
| Classification of acute leukemia | |  |  |  |  |  |
| B-ALL | 25.6 | 1.472 | 0.235 | 7.8 | 0.103 | 0.902 |
| T-ALL | 23.1 |  |  | 7.4 |  |  |
| AML | 27.3 |  |  | 7.8 |  |  |
| Time since diagnosis | |  |  |  |  |  |
| ≤ 1 month | 24.8 | 3.553 | **0.047** | 8 | 0.163 | 0.85 |
| > 1-6 months | 24.8 |  |  | 7.9 |  |  |
| > 6 months-1 year | 27 |  |  | 8.1 |  |  |
| > 1-5 years | 25.6 |  |  | 7 |  |  |
| BMI classification |  |  |  |  |  |  |
| Underweight | 22 | 0.987 | 0.402 | 6.8 | 0.379 | 0.768 |
| Normal | 25.6 |  |  | 7.9 |  |  |
| Overweight | 26.1 |  |  | 7.5 |  |  |
| Obese | 28.3 |  |  | 8.3 |  |  |
| Total hospital visits after diagnosis | | | |  |  |  |
| <5 times | 24.2 | 4.814 | **0.016** | 7.9 | 0.163 | 0.85 |
| 5-10 times | 26.4 |  |  | 7.8 |  |  |
| >10 times | 27.2 |  |  | 7.5 |  |  |
| Days interval from diagnosis to starting chemotherapy | | | |  |  |  |
| ≤3 days | 21.5 | 6.545 | **0.002** | 7.6 | 3.917 | **0.023** |
| 4-7 days | 24.8 |  |  | 9.3 |  |  |
| >7 days | 30.4 |  |  | 5.9 |  |  |
| Duration of chemotherapy | | |  |  |  |  |
| ≤6 months | 24.6 | 0.923 | 0.433 | 7.9 | 2.869 | **0.046** |
| >6-12 months | 27.5 |  |  | 8.3 |  |  |
| >12-24 months | 25.9 |  |  | 7.3 |  |  |
| >24 months | 25.1 |  |  | 6.4 |  |  |
| Current stage of the latest chemotherapy | | |  |  |  |  |
| Induction | 24.3 | 1.086 | 0.341 | 7.8 | 0.111 | 0.895 |
| Consolidation | 25.4 |  |  | 8.1 |  |  |
| Intensification | 26.6 |  |  | 7.6 |  |  |
| Pre-admission treatment | |  |  |  |  |  |
| Yes | 26.1 | 0.566 | 0.573 | 8.1 | 0.861 | 0.391 |
| No | 25.2 |  |  | 7.6 |  |  |

*x̄*; mean, *t*; t-test statistic for two groups, *F*; F-test statistic for more than two groups, *P*-value ≤ 0.05 indicates statistical significance
