# Supplementary material for: A multicenter cross-sectional study on factors associated with caregiving appraisal in pediatric acute leukemia caregivers
Source: PLoS One. 2025 Jun 6;20(6):e0324589. doi: 10.1371/journal.pone.0324589 (PMC12143579; doi:10.1371/journal.pone.0324589)
Supplement: S5 Table — *; p < .05, **; p < .001. (DOCX) [file pone.0324589.s005.docx]

**S5 Table. Correlation Matrix of Patient Symptoms and Caregiving Appraisal**

|  | Burden | Satisfaction | Mastery | Environment |
| --- | --- | --- | --- | --- |
| Anxiety about surgical procedures | 0.081 | -0.296** | -0.212* | 0.259** |
| Anxiety about treatment | 0.158 | -0.132 | -0.083 | 0.333** |
| Cognitive impairment | 0.085 | -0.015 | 0.160 | 0.009 |
| Communication difficulties | 0.210* | -0.110 | 0.01 | 0.109 |
| Nausea and vomiting | 0.120 | -0.100 | -0.005 | -0.036 |
| Pain (5-1) | 0.158 | -.334** | -0.210* | 0.088 |
| Worry | 0.237* | 0.019 | 0.154 | 0.192 |

*p < .05, **p < .001
